# Efficacy and Safety of Endovascular Treatment for Acute Large-Vessel Ischemic Stroke Beyond 6 h After Symptom Onset: A Meta-Analysis

**DOI:** 10.3389/fneur.2021.654816

**Published:** 2021-05-28

**Authors:** Ye Zhongxing, Liu Zhiqiang, Wang Jiangjie, Chen Qing, Zhang Jinfeng, Weng Chaoqun, Li Feng

**Affiliations:** ^1^Department of Neurosurgery, Fujian Sanbo Funeng Brain Hospital, Fuzhou, China; ^2^Department of Neurosurgery, Linyi Central Hospital, Linyi, China; ^3^Department of Pathology, Linyi Central Hospital, Linyi, China; ^4^Department of Neurosurgery, Fujian Medical University Union Hospital, Fuzhou, China

**Keywords:** delayed presentation, endovascular treatment, ischemic stroke, time-to-treatment, meta-analysis

## Abstract

**Background:** There is considerable evidence on the benefits of endovascular thrombectomy (EVT) for acute ischemic stroke (AIS) within 6 h after symptom onset. However, uncertainties remain regarding EVT efficacy beyond 6 h after symptom onset. We undertook a meta-analysis to assess the efficacy and safety of EVT in patients with AIS >6 h after symptom onset.

**Methods:** We searched PubMed, EMBASE, and Chinese Biomedical through July 2019. We included studies involving early (≤6 h) vs. delayed (>6 h) EVT in selected patients with AIS, based on radiological evaluation criteria. Functional independence, successful recanalization, mortality, and symptomatic intracranial hemorrhage (sICH) rates were assessed.

**Results:** Eight articles, with 3,265 patients who had undergone early EVT and 1,078 patients who had received delayed EVT, were included in the meta-analysis. Patients treated with early EVT showed a similar proportion of functional independence at 90 days [odds ratio (OR) = 1.14, 95% confidence interval (CI) = 0.926–1.397, *P* = 0.219; *I*^2^ = 36.2%, *P* = 0.128] as those treated with delayed EVT. Delayed EVT was also associated with no significant difference in mortality (OR = 1.015, 95% CI = 0.852–1.209; *P* = 0.871; *I*^2^ = 0.0%, *P* = 0.527), successful recanalization (OR = 1.255, 95% CI = 0.923–1.705; *P* = 0.147; *I*^2^ = 60.5%, *P* = 0.009), and sICH (OR = 0.976, 95% CI = 0.737–1.293; *P* = 0.871; *I*^2^ = 0.0%, *P* = 0.742) rates compared with early EVT.

**Conclusions:** Among selected patients with AIS, delayed EVT showed comparable outcomes in functional independence, recanalization, mortality, and sICH rates compared with early EVT.

## Introduction

Acute ischemic stroke (AIS) is one of the leading causes of mortality and long-term disability globally (1). In recent years, overwhelming evidence has demonstrated the benefits of endovascular thrombectomy (EVT) for AIS within 6 h after symptom onset (2). Six randomized controlled trials (RCTs) (MR CLEAN, ESCAPE, EXTEND-IA, REVASCAT, SWIFT-PRIME, and THRACE) concerning EVT for large vessel occlusion (LVO) in the anterior circulation established this therapy as a new standard for AIS treatment (1–3). However, uncertainties remain regarding the efficacy and safety of EVT for patients with AIS and LVO when performed >6 h after symptom onset (4). Recent American Heart Association/American Stroke Association (AHA/ASA) and European Stroke Organization (ESO) guidelines have recommended that EVT should be performed within 6 h of symptom onset for patients with AIS (5, 6).

However, notably, two recent RCTs, namely, the DAWN trial and the DEFUSE trial, have challenged previous understandings. In patients with a mismatch between deficit and infarct, the DAWN trial demonstrated that EVT was beneficial 6–24 h after symptom onset when compared with standard medical care only (7). Perfusion or advanced computed tomography (CT) perfusion was also a criterion for patient selection in the DEFUSE-3 trial (8). The results indicated that EVT plus standard medical care for patients with AIS 6–16 h after symptom onset was associated with more favorable efficacy and safety than standard medical therapy alone (8). The aforementioned two RCTs emphasized the importance of the tissue window in saving potentially salvageable brain tissue. Hacke (9) reported that the usual 6-h time window for EVT should be replaced with a “tissue window.” These findings would appear to indicate that the current time window needs to be reconsidered.

Furthermore, prior to these RCTs, several studies had challenged the 6-h time window. Some retrospective studies have shown that patients with AIS who had been treated with delayed EVT (>6 h after symptom onset) showed comparable efficacy and safety compared with patients with AIS who had been treated with early EVT (within 6 h after symptom onset) (10, 11). These studies provided further evidence for delayed EVT in patients with AIS that needs consideration. Therefore, we undertook a meta-analysis to assess the efficacy and safety of EVT for patients with AIS who had been treated >6 h after symptom onset.

## Methods

The protocol for this meta-analysis followed the PRISMA (Preferred Reporting Items for Systematic Reviews and Meta-Analyses) guidelines (12).

### Search Strategy

We searched PubMed, EMBASE, and Chinese Biomedical (CBM) databases for relevant articles up until July 2019, using the following search terms: “reperfusion OR recanalization OR mechanical thrombectomy OR endovascular thrombectomy OR endovascular treatment” AND “stroke OR acute ischemic stroke OR ischemic stroke OR cerebrovascular accident” AND “extend OR delay OR beyond OR more than 6.” In addition, references within included studies and relevant review articles were also screened to avoid missing potentially eligible studies.

### Eligibility Criteria

Study inclusion criteria were as follows: (1) studies concerning early EVT (within 6 h after symptom onset) vs. delayed (>6 h after symptom onset); (2) studies concerning non-contrast CT, CT angiography (CTA), CT perfusion, or perfusion–diffusion magnetic resonance imaging (MRI) used to select patients (aged ≥18 years) regardless of the time window; (3) studies in which the stroke location of the included patients comprised anterior and posterior circulation occlusion; and (4) studies that included the following data: (i) functional outcomes using the modified Rankin scale (mRS) at 90 days, (ii) mortality at 90 days, (iii) successful recanalization (modified thrombolysis in cerebral infarction and the modified treatment in cerebral infarction (mTICI) score, ≥2b), and (iv) symptomatic intracranial hemorrhage (sICH) at 90 days.

### Study Selection

Two investigators (ZXY and JJW) independently screened the study titles and abstracts. After the initial screening and selection, the full texts of the remaining articles were assessed for further processing. Disagreements were discussed and resolved with the help of the senior investigator (FL).

### Data Extraction

ZQL, QC, JFZ, and CQW independently extracted data from the primary text of the included studies and Supplementary Materials. These data included author name, year of publication, number of patients (including cases and controls), mechanical thrombectomy devices, cutoff interval (early EVT vs. delayed EVT), mean age, number of EVT combined with intravenous tissue plasminogen activator, stroke location, efficacy outcomes (functional independence, mRS score 0–2; successful recanalization, mTICI score 2b−3), and safety outcomes (sICH and mortality).

### Outcome Measures

The primary efficacy outcome was the proportion of patients with functional independence (mRS score, 0–2) at 90 days. The secondary efficacy outcome was the proportion of patients who achieved successful recanalization (mTICI score, 2b−3). The primary safety outcome was all-cause mortality at 90 days. The secondary safety outcome was the ratio of patients with sICH at 90 days.

### Statistical Analysis

Statistical analysis was performed using STATA 12 software to establish a random-effects model (because of possible heterogeneity among studies) for each outcome. The pooled odds ratios (ORs) with corresponding 95% confidence intervals (CIs) were calculated. Primary and secondary outcomes were then assessed via the pooled ORs weighted using inverse variance. The heterogeneity among studies was measured using the *I*^2^-value (significance set at >50%) (13). Sensitivity and subgroup analyses were used to determine potential influencing factors. Begg's funnel plot analysis and Egger's test were used to estimate publication bias (significance set at *P* < 0.1).

## Results

### Search Results and Study Characteristics

**Figure 1:**
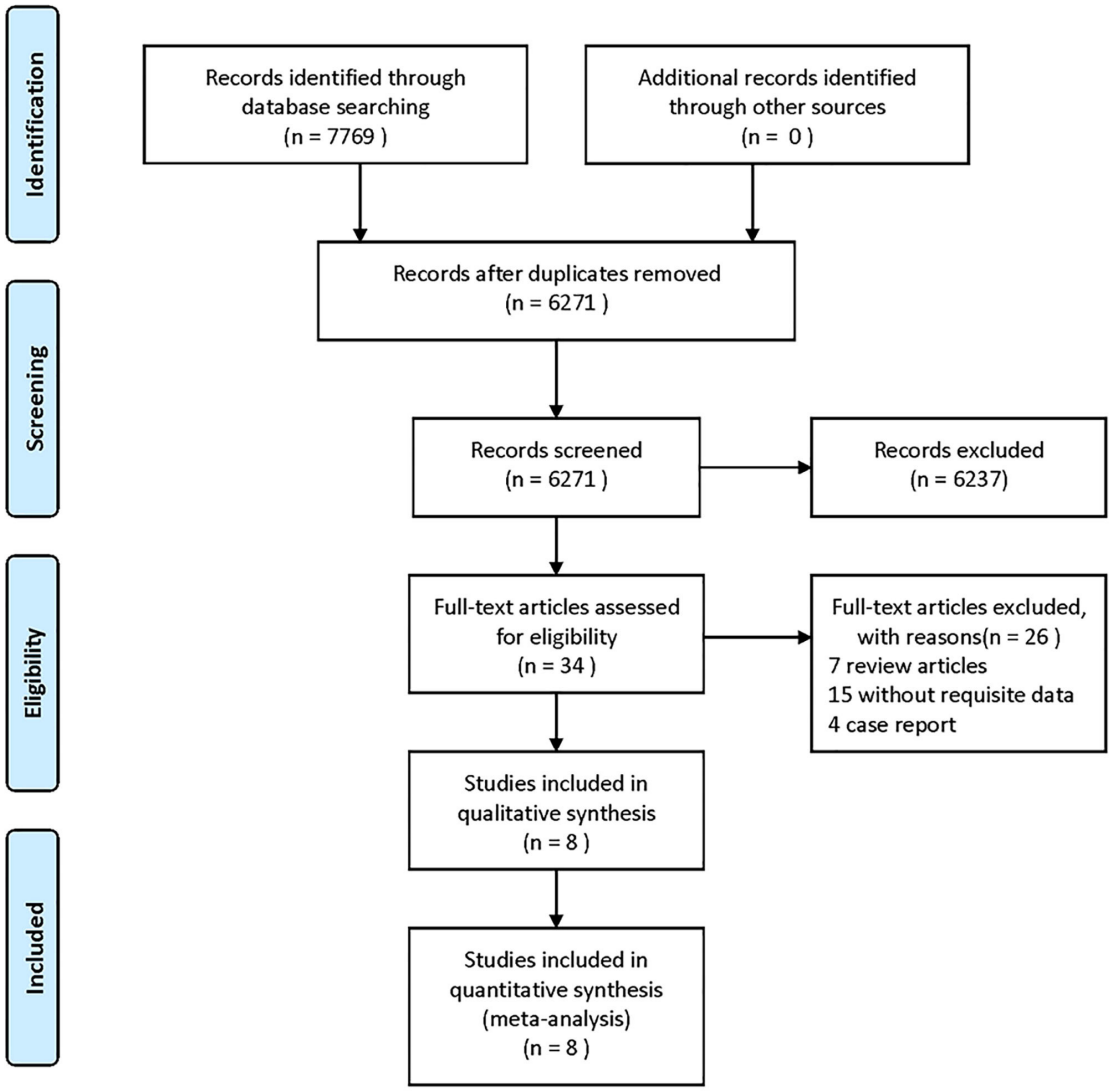
Flow diagram of the study selection process.

Figure 1 shows the search and selection process used in this meta-analysis. Our search identified 7,769 studies from PubMed, Web of Science, and CBM databases (Figure 1). After removing duplicates and ineligible articles, 34 articles were eligible for further assessment. After a full-text review, 26 articles were excluded. Data were extracted from eight eligible articles [including nine studies; the same data analyzed with different cutoff intervals were considered as different studies, namely, Mokin et al. (14), Meinel et al. (11), Motyer et al. (10), Motyer et al.^*^ (10), Millán et al. (15), Turk et al. (16), Turk et al. (17), Jung et al. (18), and Abou-Chebl et al. (19)].

In these nine studies, a total of 4,343 patients (mean age, 59.4–74 years) were included in the meta-analysis. Of these, 3,265 patients with AIS had undergone early EVT, and 1,078 patients with AIS had received delayed EVT. Detailed data concerning the study patients' characteristics are summarized in Table 1.

**Table 1:** Characteristics of studies included in the meta-analysis.

**References**	**No. of patients**	**Devices**	**Cut-off interval**	**Early EVT, NO (%)**				**Delayed EVT, NO (%)**			
				**Age (mean ± SD)**	**No. of patients**	**IV t-PA No. (%)**	**Stroke location**	**Age (mean ± SD)**	**No. of patients**	**IV t-PA**	**Stroke location**
Mokin et al. (14)	807	Solitaire FR Trevo device	6 h	67.4 ± 14.9	559	150 (26.9%)	MCA M1&M2: 295 (53.1%) MCA M3: 128 (23%) ACA: 3 (0.5%) ICA: 130 (23.4%)	66.1 ± 14.6	248	48 (19.4%)	MCA M1&M2: 144 (58.3%) MCA M3: 56 (22.7%) ACA: 0 (0%) ICA: 47 (19%)
Meinel et al. (11)	1,461	Solitaire FR	6 h	73.8	1,068	614 (57.5%)	MCA M1: 644 (60.2%) MCA M2: 138 (12.9%) Carotid-T/L: 244 (22.8%) Intracranial ICA: 34 (3.2%)	74	393	130 (33.1%)	MCA M1: 229 (58.3%) MCA M2: 51 (13%) Carotid-T/L: 95 (24.2%) Intracranial ICA: 18 (4.6%)
Motyer et al. (10)	355	Stent retrievers	6 h	68 ± 14	281	177 (63%)	Anterior circulation	68 ± 14	74	32 (43%)	Anterior circulation
Motyer^*^ et al. (10)	355	Stent retrievers	7.3 h	NA	317	NA	Anterior circulation	NA	38	NA	Anterior circulation
Millán et al. (15)	141	Solitaire FR Trevo device Merci retriever	6 h 10 min	66.5 ± 12.1	109	68 (62.4%)	MCA M1: 63 (57.8%) MCA M2: 9 (8.3%) TICA: 18 (16.5%) ICA: 4 (3.7%) TANDEM: 15 (13.8%)	64.7 ± 13.1	32	3 (9.4%)	MCA M1: 13 (40.6%) MCA M2: 2 (6.3%) TICA: 7 (21.9%) ICA: 2 (6.3%) TANDEM: 8 (25%)
Turk et al. (16)	140	Penumbra Balloon angioplasty Merci retrieval system	7 h	68	70	33 (46.5%)	ICA: 18 (25.4%) MCA: 45 (64.8%) Posterior circulation: 7 (9.9%)	64.9	70	21 (30.4%)	ICA: 11 (15.9%) MCA: 46 (65.2%) Posterior circulation: 13 (18.8%)
Turk et al. (17)	247	Penumbra aspiration system.	8 h	67	173	95 (55.2%)	Anterior circulation: 158 (92.4%) Posterior circulation: 15 (8.6%)	64	74	20 (27%)	Anterior circulation: 61 (83.6%) Posterior circulation: 13 (16.4%)
Jung et al. (18)	782	Aspiration Solitaire stent PTA Other retriever	6 h	63.3 ± 13.5	654	547 (83.7%)	Carotid artery: 160 (24.5%) MCA: 419 (64.1%) Posterior cerebral artery: 9 (1.4%) Anterior cerebral artery: 5 (0.8%) Basilar/vertebral artery: 61 (9.3%)	61.1 ± 15.1	128	97 (75.8%)	Carotid artery: 36 (28.1%) MCA: 43 (33.6%) Posterior cerebral artery: 5 (3.9%) Anterior cerebral artery: 1 (0.8%) Basilar / vertebral artery: 43 (33.6%)
Abou-Chebl (19)	55	Merci Retriever	6 h	63.4 ± 16.2	34	20 (58.8%)	MCA: 15 (44.1%) ICA: 4 (11.8%) TANDEM: 11 (32.4%) Vertebrobasilar: 4 (11.8%)	59.4 ± 17.2	21	5 (23.8%)	MCA: 8 (38.1%) ICA: 4 (19%) TANDEM: 11 (9.5%) Vertebrobasilar: 7 (33.3%)

***IV t-PA**, intravenous thrombolysis with tissue plasminogen activator; **EVT**, endovascular treatment; **ICA**, internal carotid artery; **ACA**, anterior cerebral artery; **MCA**, middle cerebral artery; **TICA**, terminal internal carotid artery; **PTA**, percutan transluminal angioplasty*.

### Quantitative Synthesis

**Figure 2:**
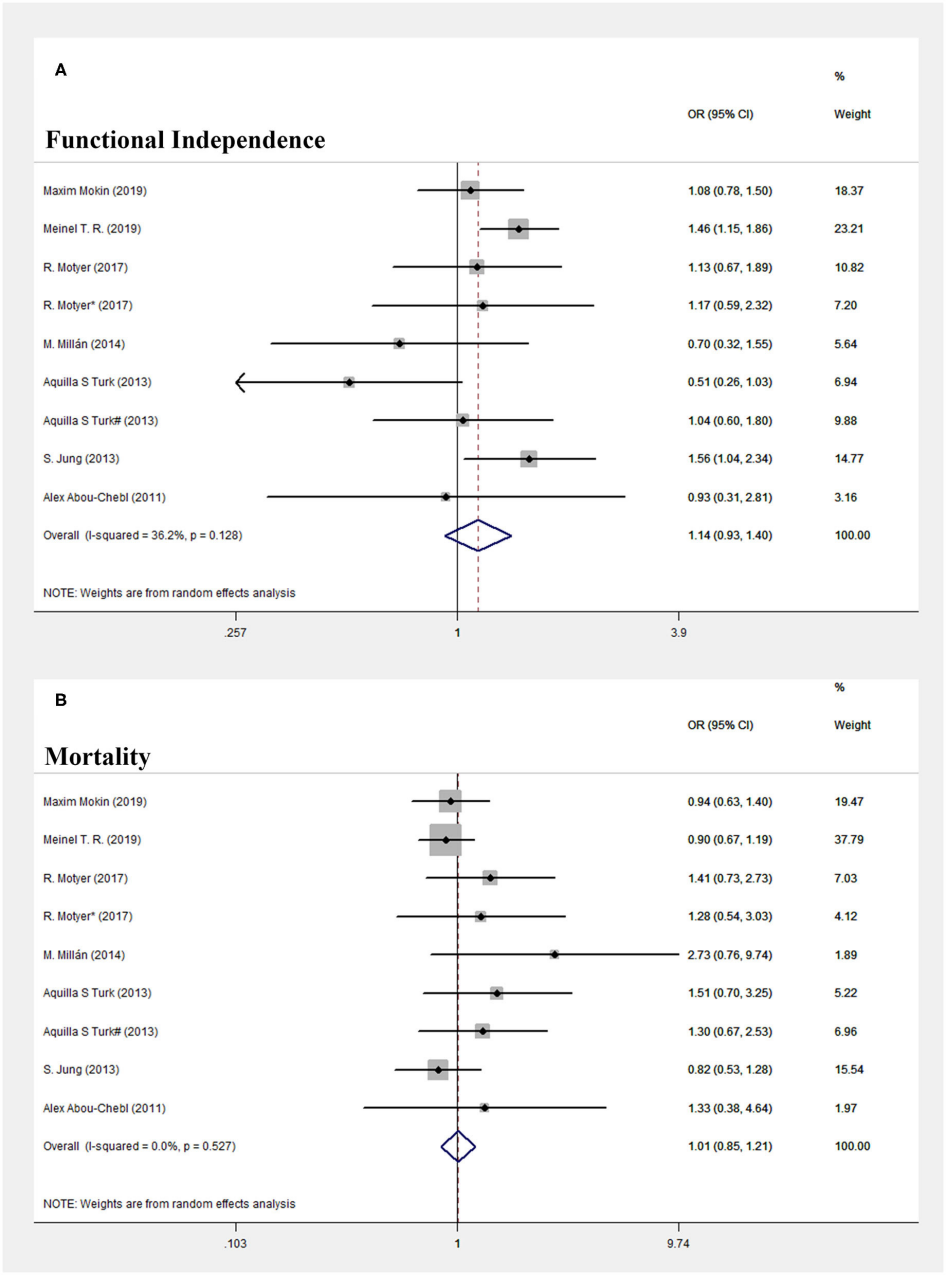
Forest plot of comparison. **(A)** Functional independence. **(B)** Mortality.

In terms of primary efficacy outcomes, patients treated with early EVT showed a similar proportion of favorable functional outcome at 90 days compared with those treated with delayed EVT (OR = 1.14, 95% CI = 0.926–1.397, *P* = 0.219; *I*^2^ = 36.2%, *P* = 0.128, respectively, Figure 2A).

In terms of primary safety outcomes, no significant difference in mortality rates within 90 days was found between the early and delayed EVT patient groups (OR = 1.015, 95% CI = 0.852–1.209, *P* = 0.871; *I*^2^ = 0.0%, *P* = 0.527, respectively, Figure 2B).

**Figure 3:**
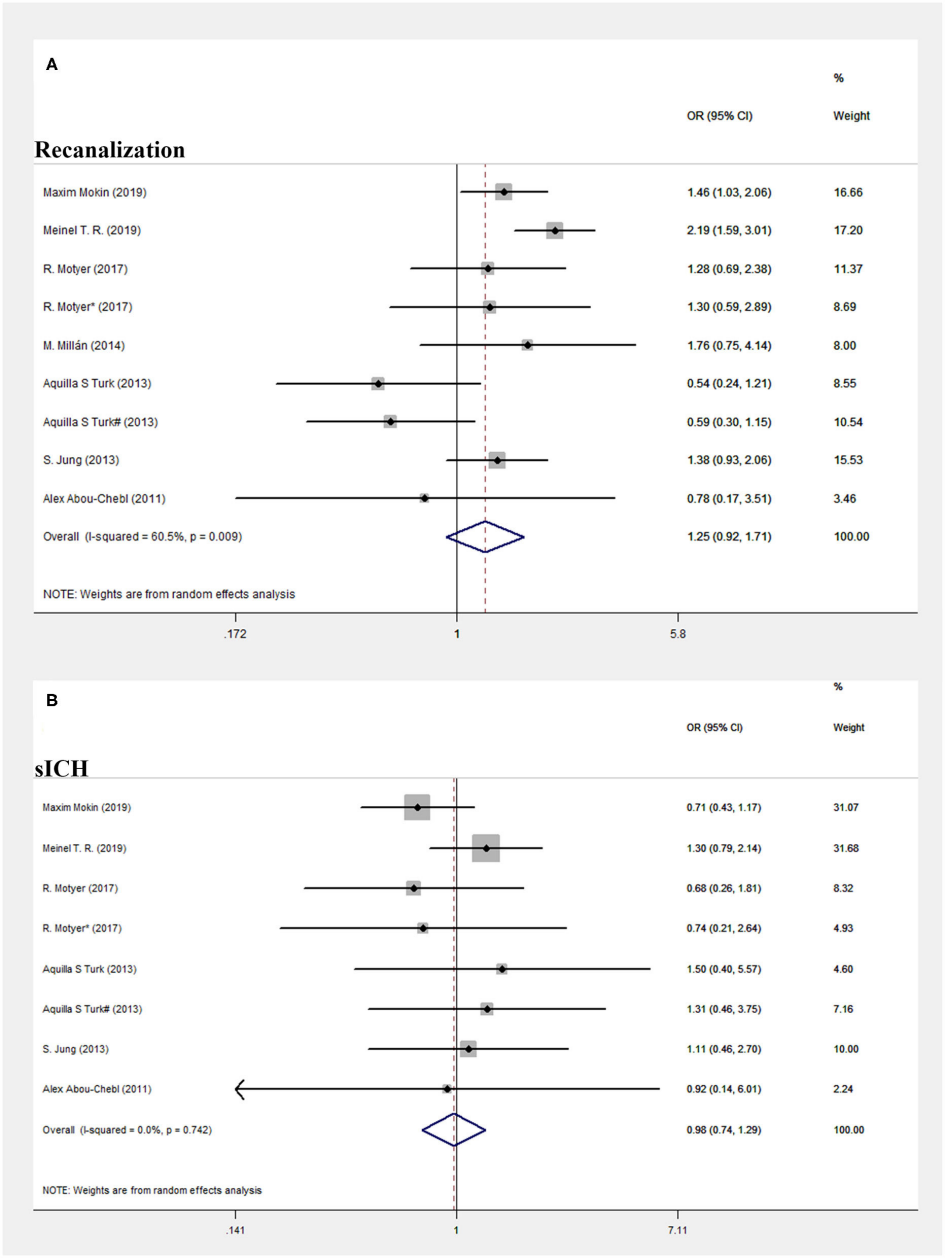
Forest plot of comparison. **(A)** Successful recanalization. **(B)** Systematic intracranial hemorrhage.

In terms of secondary efficacy outcomes, the rates of successful recanalization (mTICI score, 2b−3) were 2,645/3,265 (81%) and 821/1,077 (76.2%) in the early and delayed EVT patient groups, respectively (OR = 1.255, 95% CI = 0.923–1.705, *P* = 0.147; *I*^2^ = 60.5%, *P* = 0.009, respectively; Figure 3A), but the differences between the groups were not statistically significant. However, there was significant heterogeneity in the secondary efficacy outcomes (*I*^2^ = 60.5%, *P* = 0.009). Thus, we performed subgroup analysis to find out the potential influencing factors. As statistical heterogeneity was revealed in the forest plot showing non-overlapping CIs for all the included studies, the eligible studies were divided into “overlapping CI” and “non-overlapping CI” group. As shown in Supplementary Figure 1, *I*^2^ = 36.0%, *P* = 0.154, in the “overlapping” group, and *I*^2^ = 65.0%, *P* = 0.091, in the “non-overlapping” group. The heterogeneity was decreased after grouping. Interestingly, we found a common feature in these two studies (Maxim Mokin-2019 and Meinel T. R.-2019) in “non-overlapping CI” group. Both studies performed all thrombectomies by using Solitaire FR Revascularization Device with or without Trevo device. As we know, devices for mechanical thrombectomy were important factors associated with improved functional outcomes and rate of recanalization (2). Hence, the devices for thrombectomy may be one of the potential influencing factors of heterogeneity.

In terms of secondary safety outcomes, there was also no significant difference in the rates of sICH within 90 days between the patient groups: 6.67% for early EVT and 7.09% for delayed EVT (OR = 0.976, 95% CI = 0.737–1.293, *P* = 0.871; *I*^2^ = 0.0%, *P* = 0.742, respectively, Figure 3B). Detailed data concerning efficacy and safety outcomes are summarized in Table 2.

**Table 2:** The detailed date of efficacy and safety outcome.

**References**	**mRS0-2**				**Mortality**				**Successful recanalization**				**Symptomatic ICH**			
	**Eevent**	**Enon-event**	**Devent**	**Dnon-event**	**Eevent**	**Enon-event**	**Devent**	**Dnon-event**	**Eevent**	**Enon-event**	**Devent**	**Dnon-event**	**Eevent**	**Enon-event**	**Devent**	**Dnon-event**
Mokin et al. (14)	234	252	96	112	100	386	45	163	444	115	180	68	44	507	27	220
Meinel et al. (11)	463	605	135	258	209	859	84	309	961	107	316	77	73	995	21	372
Motyer et al. (10)	130	151	32	42	65	216	13	61	228	53	57	17	16	265	6	68
Motyer et al.^*^ (10)	146	171	16	22	71	246	7	31	256	61	29	9	19	298	3	35
Millán et al. (15)	45	64	16	16	24	85	3	29	84	25	21	11	11	98	0	32
Turk et al. (16)	21	50	31	38	21	50	15	54	51	20	57	12	6	65	4	65
Turk et al. (17)	74	99	31	43	43	130	15	59	124	49	60	14	15	158	5	69
Jung et al. (18)	294	346	43	79	145	495	32	90	469	184	83	45	34	618	6	121
Abou-Chebl (19)	14	20	9	12	10	24	5	16	28	6	18	3	3	31	2	19

* *These data were reanalyzed using the 7.3-h threshold. Eevent, Early EVT events; Enon-event, Early EVT non-event; Devent, Delayed EVT event; Dnon-event, Delayed EVT non-event*.

### Publication Bias

**Figure 4:**
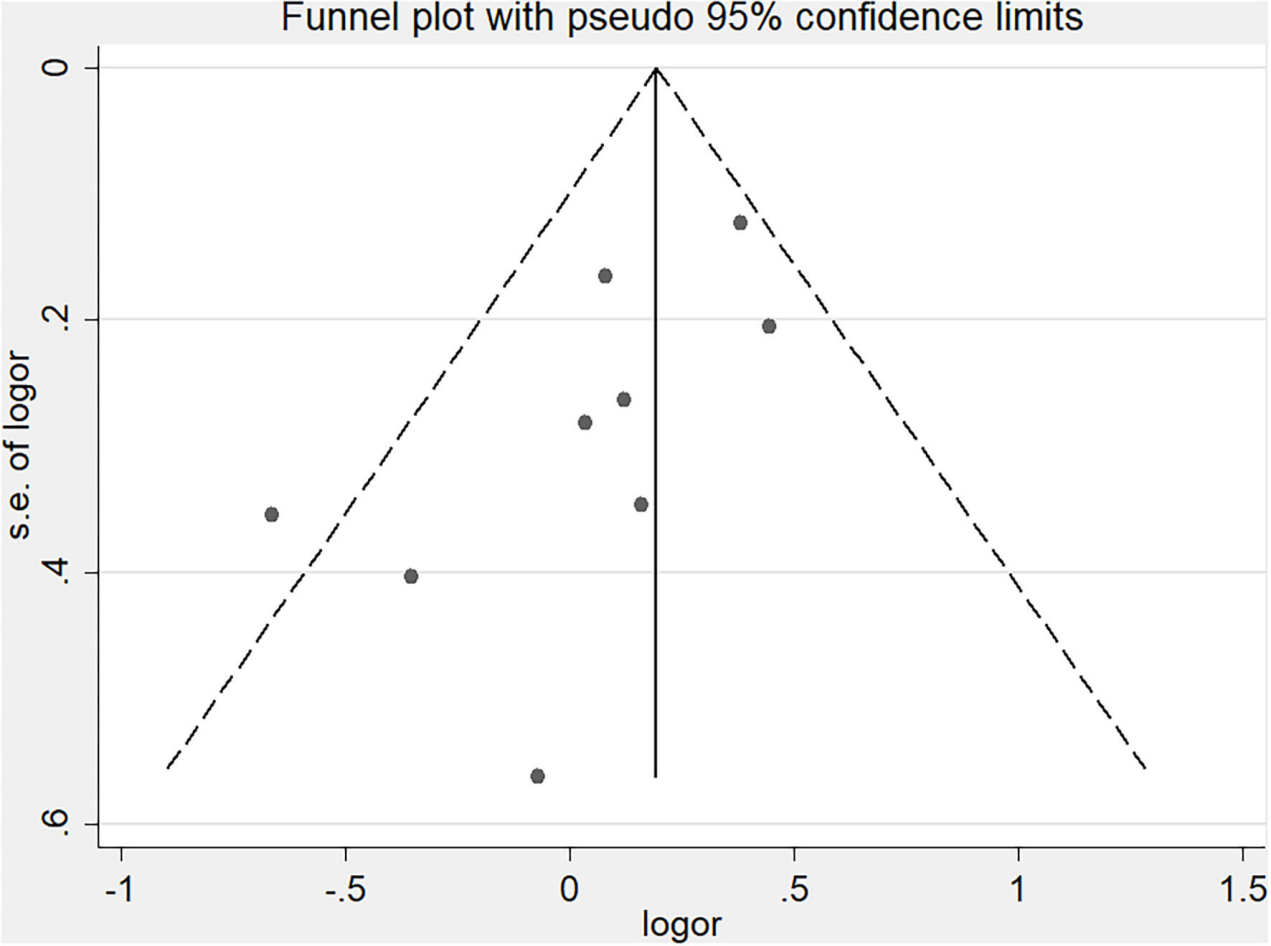
Funnel plot of included studies.

Publication bias was assessed using Egger's test and Begg's funnel plot. However, Begg's test (*P* = 0.144) and Egger's test (*P* = 0.042) showed opposite results, with Begg's funnel plot (Figure 4) indicating that publication bias might exist (funnel plot asymmetry). Therefore, we applied the trim-and-fill method to further assess whether publication bias existed (20, 21). The results indicated no significant difference before and after four iterations (Supplementary Figure 2, *Z* = 4.048, *P* < 0.001, vs. Z = 2.584, *P* = 0.01).

## Discussion

The present meta-analysis of pooled data indicated that patients with AIS who had delayed EVT treatment (>6 h) had similar rates of functional outcomes when compared with patients who received early EVT treatment (>6 h). All patients underwent imaging selection rather than guided time selection. There were no significant differences in mortality, recanalization, and sICH rates between the early and delayed EVT patient groups.

Six previous RCTs, namely, MR CLEAN, ESCAPE, EXTEND-IA, REVASCAT, SWIFT-PRIME, and THRACE, involving EVT for LVO in the anterior circulation established this therapy as a new standard of AIS treatment (1–3, 22). These RCTs showed that EVT treatment was associated with improved functional outcomes within 6 h after symptom onset when compared with standard medical care in patients with AIS. Following this “revolution” in stroke treatment, AHA/ASA, and ESO guidelines recommended that EVT should be performed within 6 h of symptom onset for patients with AIS (5, 6). However, this specific therapeutic window has been contested. The HERMES study found that EVT therapy was effective within 7.3 h after stroke onset and less effective beyond 7.3 h (4). The DAWN and DEFUSE-3 trials showed that EVT is beneficial 6–24 h after symptom onset in selected patients with proximal vessel occlusion in the anterior circulation (7, 8). These results indicate that a firm time window for stroke treatment may be obsolete and that the role of the time window needs to be reexamined (23). Time may not be a less critical factor than previously thought in independently affecting the prognosis of patients with AIS. Nogueira et al. reported that time (to treatment or to reperfusion) was one of many variables that may influence the outcomes of proximal vessel occlusion for patients with stroke and that it appeared to show a more modest effect during the later phases of stroke evolution (24). Nathan et al. reported that EVT for patients with AIS >24 h from symptom onset was safe and effective; however, further evidence-based trials to evaluate benefits vs. risks using prolonged time windows are required (25).

In contrast to the DAWN and DEFUSE-3 trials, we pooled data from several retrospective studies that had directly compared early and delayed EVT. In terms of primary efficacy outcomes, patients treated with early EVT showed a comparable proportion of favorable functional outcomes at 90 days compared with those treated with delayed EVT. Furthermore, primary safety outcomes also showed no significant difference in 90-day mortality rates between the early and delayed EVT patient groups. These results indicated comparable outcomes in both groups in terms of effectiveness and safety. Our findings provided further evidence supporting delayed EVT as being a beneficial intervention in appropriately selected patients with AIS. Therefore, it is vital to identify patients with salvageable brain tissue, and aggressive endovascular treatment should be encouraged.

Our study has several limitations. The criteria for patient selection were heterogeneous (based on nonc-ontrast CT, CTA, CT perfusion, or perfusion–diffusion MRI). The studies in our meta-analysis included patients with both anterior and posterior circulation occlusion. The original study data were not available; therefore, more subgroup analyses could not be performed, which led to the possibility of data heterogeneity. Although the present study showed a comparable result in functional independence between early and delayed EVT group, mild heterogeneity still existed (*I*^2^ = 36.2%, *P* = 0.128). As shown in Supplementary Figure 3, the heterogeneity was eliminated after stratification (*I*^2^ = 0.0%, *P* = 0.785; *I*^2^ = 0.0%, *P* = 0.553). We found that these two studies (Meinel T. R.-2019 and S. Jung-2013) included several unclear-onset stroke or wake-up stroke patients. For this part of patients, the time from stroke onset to recanalization may be much longer than 6 h. As the time to treatment may have an important effect on the efficacy of EVT. Longer EVT procedures lead to lower rates of functional independence and higher rates of sICH 2. That may partly explain the reason of heterogeneity. However, the present study cannot exclude all the relevant affecting causes. Moreover, time to treatment may have had an important effect on the efficacy of EVT. Longer ET procedures are known to lead to lower rates of functional independence and higher rates of sICH (26). In this meta-analysis, we could not exclude relevant affecting causes. In addition, the retrospective nature of the included studies was a notable limitation in that criteria used in the selection of patients who received EVT in the early and late windows may not have been comparable, which may have affected our study findings. Hence, these results require further evaluation prior to any implementation in clinical practice.

## Conclusion

Among patients with AIS, those in the delayed EVT group (>6 h) showed comparable outcomes in functional independence and recanalization rates compared with those in the early EVT group (>6 h). There were no significant differences in mortality rates and sICH between the early and delayed EVT patient groups. These data support delayed EVT as a beneficial intervention for appropriately selected patients with AIS.

## Data Availability Statement

The original contributions presented in the study are included in the article/Supplementary Material, further inquiries can be directed to the corresponding author/s.

## Author Contributions

YZ, LZ, and WJ developed the study protocol and drafted the manuscript. CQ, ZJ, and WC analyzed the data and performed a meta-analysis. LF revised the manuscript and edited the language. All authors contributed to the article and approved the submitted version.

## Conflict of Interest

The authors declare that the research was conducted in the absence of any commercial or financial relationships that could be construed as a potential conflict of interest.
