# Supplementary figures and images for: Efficacy and Safety of Endovascular Treatment for Acute Large-Vessel Ischemic Stroke Beyond 6 h After Symptom Onset: A Meta-Analysis
Source: Front Neurol. 2021 May 28;12:654816. doi: 10.3389/fneur.2021.654816 (PMC8195613; doi:10.3389/fneur.2021.654816)

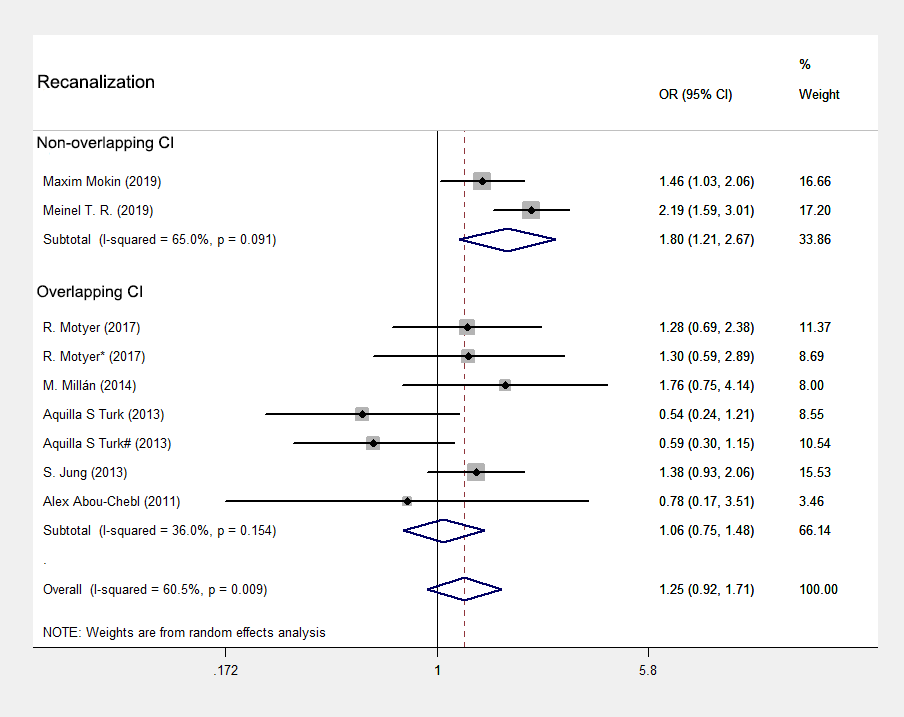

Supplement: Supplementary file 1 [file Image_1.TIF]

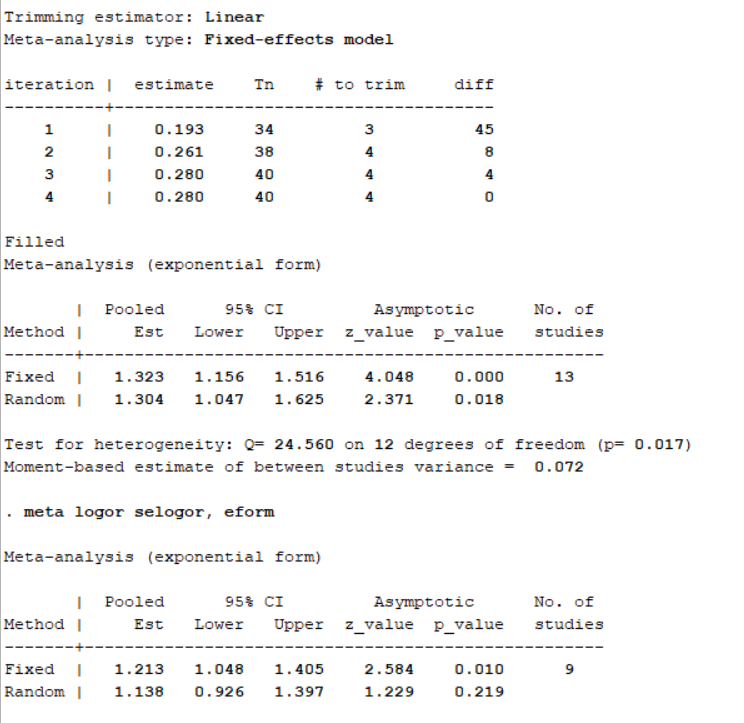

Supplement: Supplementary file 2 [file Image_2.PNG]

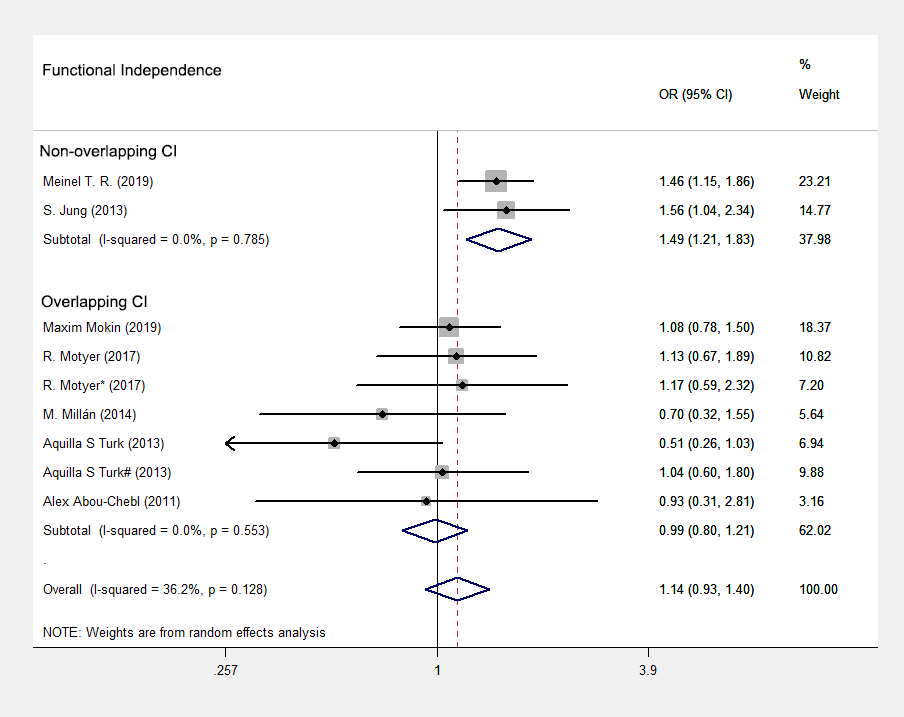

Supplement: Supplementary file 3 [file Image_3.TIF]
